# The Left Atrial Area Derived Cardiovascular Magnetic Resonance Left Ventricular Filling Pressure Equation Shows Superiority over Integrated Echocardiography

**DOI:** 10.3390/medicina59111952

**Published:** 2023-11-04

**Authors:** Ciaran Grafton-Clarke, Gareth Matthews, Rebecca Gosling, Peter Swoboda, Alexander Rothman, Jim M. Wild, David G. Kiely, Robin Condliffe, Samer Alabed, Andrew J. Swift, Pankaj Garg

**Affiliations:** 1 Department of Cardiology, Norfolk and Norwich University NHS Foundation Trust, Norwich NR4 7UY, UK; ciarang-c@hotmail.com (C.G.-C.);; 2 School of Medicine, University of East Anglia, Norwich NR4 7TJ, UK; 3 Department of Infection, Immunity and Cardiovascular Disease, The University of Sheffield, Sheffield S10 2TN, UKrobin.condliffe@nhs.net (R.C.);; 4 Division of Biomedical Imaging, Leeds Institute of Cardiovascular and Metabolic Medicine, University of Leeds, Leeds LS2 9JT, UK; 5 NIHR Biomedical Research Centre, Sheffield, S10 2JF, UK

**Keywords:** cardiovascular magnetic resonance, heart failure, echocardiography, haemodynamic assessment, filling pressure

## Abstract

*Background and objectives*: Evaluating left ventricular filling pressure (LVFP) plays a crucial role in diagnosing and managing heart failure (HF). While traditional assessment methods involve multi-parametric transthoracic echocardiography (TTE) or right heart catheterisation (RHC), cardiovascular magnetic resonance (CMR) has emerged as a valuable diagnostic tool in HF. This study aimed to assess a simple CMR-derived model to estimate pulmonary capillary wedge pressure (PCWP) in a cohort of patients with suspected or proven heart failure and to investigate its performance in risk-stratifying patients. *Materials and methods*: A total of 835 patients with breathlessness were evaluated using RHC and CMR and split into derivation (85%) and validation cohorts (15%). Uni-variate and multi-variate linear regression analyses were used to derive a model for PCWP estimation using CMR. The model’s performance was evaluated by comparing CMR-derived PCWP with PCWP obtained from RHC. *Results*: A CMR-derived PCWP incorporating left ventricular mass and the left atrial area (LAA) demonstrated good diagnostic accuracy. The model correctly reclassified 66% of participants whose TTE was ‘indeterminate’ or ‘incorrect’ in identifying raised filling pressures. On survival analysis, the CMR-derived PCWP model was predictive for mortality (HR 1.15, 95% CI 1.04–1.28, *p* = 0.005), which was not the case for PCWP obtained using RHC or TTE. *Conclusions*: The simplified CMR-derived PCWP model provides an accurate and practical tool for estimating PCWP in patients with suspected or proven heart failure. Its predictive value for mortality suggests the ability to play a valuable adjunctive role in echocardiography, especially in cases with unclear echocardiographic assessment.

## 1. Introduction

Left ventricular filling pressure (LVFP) is a critical determinant of cardiac performance and a crucial parameter in establishing the diagnosis of heart failure (HF) [1]. LVFP is a determinant of cardiac output and stroke volume, as per the Frank–Starling mechanism. Maintaining normal LVFP is crucial for optimal cardiac function and patient outcomes. Elevated LVFP is often associated with symptoms of heart failure, such as dyspnea, due to increased left atrial pressure and pulmonary capillary wedge pressure. The American College of Cardiology Foundation (ACCF), American Heart Association (AHA), and Heart Failure Society of America (HFSA) guidelines emphasise that evidence supporting increased filling pressures is important for the diagnosis of heart failure if the left ventricular ejection fraction (LVEF) is >40% [2,3]. On the other hand, if LVFP is too low, this can lead to inadequate ventricular filling and reduced cardiac output, potentially resulting in symptoms of low perfusion such as fatigue and syncope.

Conventionally, the assessment of LVFP was predominantly conducted using multi-parametric transthoracic echocardiography (TTE) or, in borderline cases, diastolic stress testing and right heart catheterisation (RHC), particularly if there was a discrepancy between right and left ventricular filling pressures [4]. Pulmonary capillary wedge pressure (PCWP) is often used as an indirect estimate of LVFP since an increase in LVFP leads to an increase in left atrial pressure, which is reflected in the PCWP measurement.

Cardiovascular magnetic resonance imaging (CMR) is increasingly used in the diagnostic workflow of patients with suspected or proven HF, including detecting scars and ischaemia, revealing subendocardial defects through stress perfusion and quantifying infiltration or fibrosis during gadolinium analysis [5]. Several methods have been developed to assess diastolic function and, by extension, PCWP [6]. In a recent study by our group [7], RHC and CMR were performed within 24 h, and participants with breathlessness were split into derivation (n = 706) and validation (n = 127) cohorts, enabling the creation of a model that can derive PCWP through CMR to be constructed. Within this model, two parameters were found to be independent predictors of PCWP: left ventricular mass (LVM) and left atrial volume (LAV).

With the recognition that there is a need for practical clinical implementation, this study leverages the left atrial area (LAA), obtained from a single view (usually the four-chamber view), to develop a simplified model of deriving PCWP by using CMR in a cohort of patients with suspected or proven HF and to determine whether this model can risk stratify patients.

We hypothesise that a CMR model that leverages the left atrial area is able to accurately predict outcomes in patients with suspected or proven heart failure. 

## 2. Materials and Methods

### 2.1. Study Population

This investigation involved a well-defined cohort of 835 individuals who were referred to our research centre for the evaluation of breathlessness. The study encompassed an extensive eight-year period, commencing in 2012 and concluding in 2020. All patients underwent right heart catheterisation and CMR, with procedures performed within 24 h of each other. The inclusion criteria included the signs and symptoms of heart failure, age > 18 years, and the provision of informed consent. The exclusion criteria included pulmonary arterial hypertension (type 1) and contraindications to RHC or CMR, including claustrophobia and end-stage heart failure. Ethical approval for the research protocol was duly granted by the National Research Ethics Service, with approval reference number 16/YH/0352 (approval date 31 October 2016). 

### 2.2. Cardiac and Pulmonary Function Assessment

The comprehensive assessment of patients’ cardiac and pulmonary functions was carried out through the implementation of two pivotal diagnostic procedures: right heart catheterisation (RHC) and cardiac magnetic resonance imaging (CMR).

Right heart catheterisation (RHC): RHC was executed using a specialised balloon-tipped 7.5 French thermodilution catheter. This meticulously conducted procedure facilitated the accurate measurement of pulmonary capillary wedge pressure (PCWP) through established methodologies. Subsequently, PCWP values were subjected to rigorous averaging over multiple cardiac cycles to ensure the precision of the obtained data.

Cardiac magnetic resonance imaging (CMR): CMR investigations were conducted employing a state-of-the-art 1.5T GE HDx scanner (GE Healthcare, Milwaukee, WI, USA). The CMR imaging protocol encompassed a diverse array of sequences, including two-chamber, three-chamber, four-chamber, and short-axis cine acquisitions using a retrospective cardiac-gated multi-slice steady-state free precession sequence (TR 2.8 ms, TE 1.0 ms, flip angle 50°, field of view 48 × 43.2, 256 × 256 matrix, 125 kHz bandwidth, and slice thickness 8–10 mm). The short-axis cine images were used to obtain the left ventricular (LV) end-diastolic volume (LVEDV), the LV end-systolic volume (LVESV), the right ventricular (RV) end-diastolic volume (RVEDV), and the RV end-systolic volume (RVESV). From end-diastolic and end-systolic volumes, the LV stroke volume (LVSV), the LV ejection fraction (LVEF), the RV stroke volume (RVSV), and the RV ejection fraction (RVEF) were calculated. For the specific purpose of quantifying the dimensions of the left atrial area (LAA), the four-chamber view was diligently employed. Using a dedicated post-processing workstation, specifically the GE Advance 4.1, the delineation of the endocardial boundary of the left atrium occurred during the end-systolic phase, immediately preceding the opening of the mitral valve. It is noteworthy that this contouring process excluded the pulmonary veins and the left atrial appendage, which are deemed extraneous to the left atrial body. Additionally, segmentation of both the endocardial and epicardial boundaries within the short-axis stack was performed to derive ventricular volumes, with particular emphasis on left ventricular mass measurements during the end-diastolic phase.

A clinical evaluation using transthoracic echocardiography (TTE) was conducted within 24 h of right heart catheterisation (RHC), following local practice guidelines. Various echocardiographic parameters were measured, adhering to the minimum dataset size recommended by the British Society of Echocardiography (BSE). Left atrial pressure was estimated using the American Society of Echocardiography (ASE) algorithm, which categorises patients into normal, elevated, or uncertain left atrial pressure based on mitral inflow parameters, tissue Doppler imaging, tricuspid regurgitation velocity, and the left atrial volume (LAV) index [4].

### 2.3. Follow-Up and Cohort Stratification

Subsequent to the initial assessments, participants were diligently monitored over a mean follow-up duration of 4.0 years. Cohort stratification ensued, dividing the subjects into two distinct groups: the derivation cohort, constituting 85% of the participant pool (n = 706), and the validation cohort, encompassing 15% of the participants (n = 127). The assessment of diastolic function within the validation cohort was conducted using transthoracic echocardiography (TTE).

### 2.4. Statistical Analysis

In accordance with established clinical practice, all clinically acquired data were assumed to follow a normal distribution. Continuous variables were expressed as the mean ± standard deviation, serving as the primary metrics for their presentation. Meanwhile, categorical data were conveyed in terms of frequencies and corresponding percentages. To ascertain significant differences between continuous variables, a two-sample independent *t*-test was employed as the statistical methodology of choice. Categorical data, on the other hand, underwent scrutiny through the χ^2^ test, a robust method for assessing associations and differences among categorical variables. For the objective of comparing cardiac outputs as determined by cardiac magnetic resonance imaging (CMR) and right heart catheterization (RHC), a paired *t*-test was applied, offering a suitable framework for analysing paired continuous data.

### 2.5. Model Development and Evaluation

The dataset was stratified into two distinct cohorts: the derivation cohort, which constituted 85% of the total population (n = 706), and the validation cohort, comprising 15% of the total population (n = 127).

Within the derivation cohort, statistical analysis encompassed several phases. Firstly, uni-variate linear regression was employed to derive Pearson correlation coefficients, elucidating the relationships between individual CMR metrics and pulmonary capillary wedge pressure (PCWP) as determined via RHC. Subsequently, a multi-variate regression model was crafted to establish relationships between various CMR metrics. Furthermore, the utility of supervised machine learning penalised regression models was explored for the prediction of CMR-derived PCWP.

### 2.6. Validation and Diagnostic Performance Assessment

The final model derived from the derivation cohort was subsequently applied to the validation cohort. Receiver operating characteristic (ROC) analysis was conducted to rigorously assess the diagnostic performance of CMR-derived PCWP in the detection of elevated RHC-derived PCWP, thereby elucidating the model’s efficacy.

### 2.7. Prognostic Analysis

For the comprehensive analysis of prognosis, Kaplan–Meier analysis was undertaken to delineate survival probabilities, while Cox’s proportional hazard model was harnessed for multi-variate prognostic evaluation.

### 2.8. Software and Significance Threshold

Statistical analyses were executed using SPSS version 22 (IBM, Chicago, IL, USA), with confirmatory analyses conducted in MedCalc (MedCalc Software, Ostend, Belgium version 19.1.5). Notably, supervised machine learning penalised regression was implemented in StataIC 16.

### 2.9. Statistical Significance

Throughout all analyses, unless explicitly specified otherwise, all statistical tests were two-tailed, and statistical significance was established at a threshold of *p* < 0.05.

### 2.10. Primary Objectives

The study’s primary objectives encompassed the examination of the correlation between CMR-derived PCWP and the PCWP values ascertained via RHC. Furthermore, the study sought to rigorously assess the performance of the developed model in stratifying individuals whose TTE results yielded non-diagnostic outcomes. These outcomes were classified as either ‘indeterminate’ or ‘incorrect’ in their capacity to accurately identify individuals with elevated filling pressures during invasive assessment. Additionally, the prognostic potential of CMR-derived PCWP was scrutinised, particularly in comparison to RHC and TTE. Prognostic values were articulated as hazard ratios (HR) within Cox’s proportional hazard regression, supplemented by visual representation through Kaplan–Meier survival curves. For an exhaustive exposition of the study’s population, research methodologies, and the intricacies of the statistical analysis, we refer readers to the previously published documentation for comprehensive reference [7]. 

## 3. Results

A total of 835 participants were included in this study. Patient characteristics for the derivation cohort (n = 708) and the validation cohort (n = 127) are summarised in Table 1. A total of 60% of participants were female (n = 498). The primary diagnosis of a patient’s breathlessness was left heart disease in 60% of patients (n = 497), lung disease in 19% of patients (n = 160), and pulmonary hypertension in 21% of patients (n = 178). Of those with left heart disease, 89% had HF with preserved ejection fraction (HFpEF), and 11% had HF with reduced ejection fraction (HFrEF). 

### 3.1. Derivation Cohort

Within the derivation cohort, during univariable regression, the LAA demonstrated a moderate association with RHC PCWP (R = 0.50, 95% CI 0.44–0.55, *p* < 0.0001). When compared to all other CMR metrics previously tested, the LAA was most strongly correlated with RHC PCWP. 

**Figure 1:**
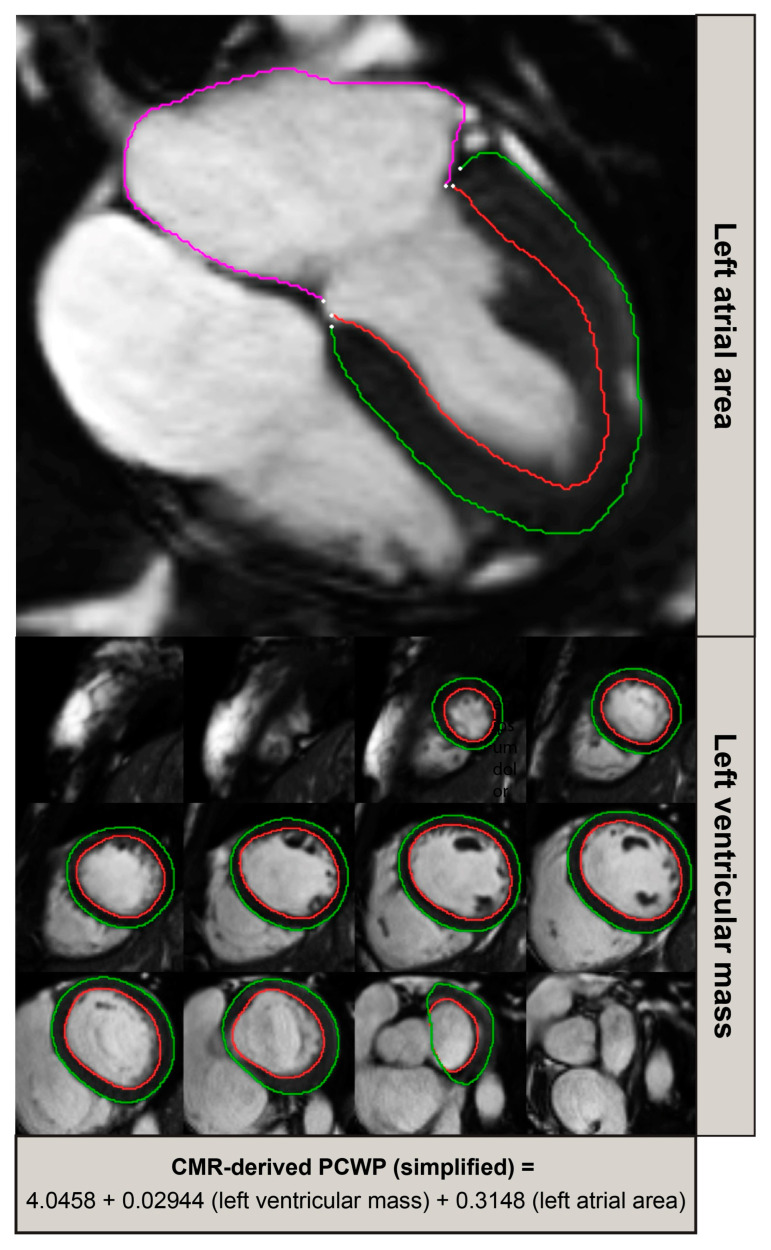
A simplified model of CMR-derived PCWP incorporating left ventricular mass and left atrial area. Red contour indicates the endocardium and the green contour indicates the epicardium. The purple contour indicate the left atrial endocardial wall.

Within the derivation cohort, during backward multi-variate regression, the two CMR variables that demonstrated an independent association with invasively measured PCWP were the LAA and LVM (Figure 1). The following equation was derived:

CMR-derived PCWP = 4.0584 (constant) + 0.3148 (LAA) + 0.02944 (LVM)

### 3.2. Validation Cohort

**Figure 2:**
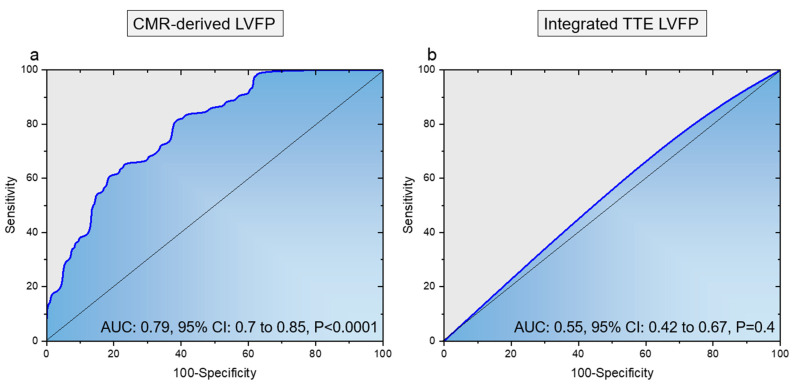
Panel (**a**): Receiver operator characteristics curve for CMR-modelling PCWP against measured PCWP 15 mmHg to identify raised left ventricular filling pressures. Panel (**b**): Receiver operator characteristics curve for integrated TTE PCWP against measured PCWP 15 mmHg to identify raised left ventricular filling pressures.

The area under the curve for the CMR-derived PCWP model was 0.79 (95% CI 0.70–0.85, *p* < 0.0001, Figure 2a). The area under the curve for integrated TTE LVFP assessment was 0.55 (95% CI 0.42–0.67, *p* = 0.4, Figure 2b).

**Figure 3:**
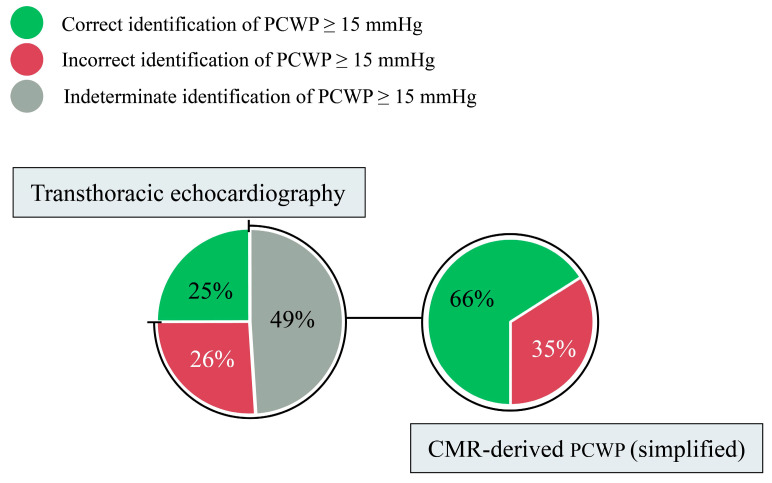
A simplified CMR-derived PCWP model correctly reclassified 66% of participants where TTE failed to identify participants with ‘elevated’ filling pressures.

Using RHC, PCWP values were obtained, where a threshold of 15 mmHg was used to differentiate ‘normal’ from ‘elevated’ filling pressures. However, when using TTE to identify ‘elevated’ filling pressure within the validation cohort, results were ‘indeterminate’ in 49% of participants and ‘incorrect’ in 26%. On applying the simplified CMR-derived PCWP model, CMR was able to accurately reclassify 66% (n = 63) of the participants who had previously been marked as ‘incorrect’ or ‘indeterminate’ using TTE (Figure 3). 

**Figure 4:**
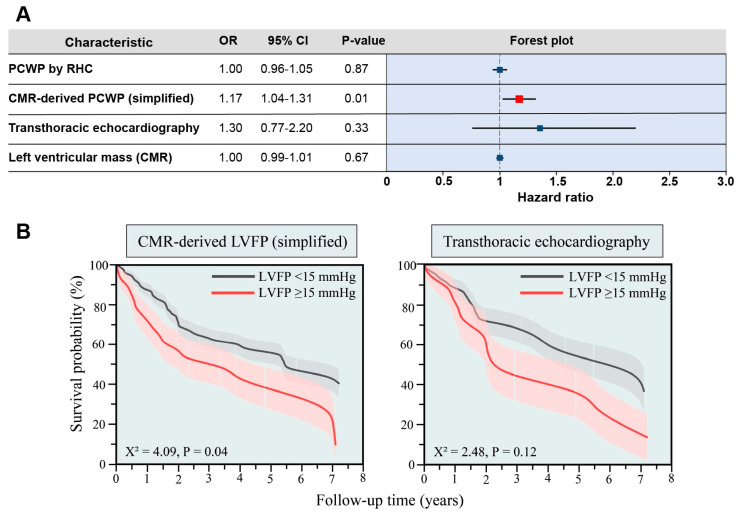
Panel (**A**): Cox’s proportional hazard regression within the validation cohort demonstrates that a simplified CMR-derived PCWP model predicts mortality, whereas predicting PCWP by RHC and TTE does not. Panel (**B**): Kaplan–Meier survival curves within the validation cohort demonstrate the prognostic significance of a simplified CMR-derived PCWP model, whereas assessment using TTE does not.

In Cox’s proportional hazard regression survival analysis (Figure 4A) within the validation cohort (mean follow-up of 5.2 ± 0.3 years), the simplified CMR-derived PCWP model was predictive for mortality (HR 1.17, 95% CI 1.04–1.31, *p* = 0.01). This was not the case for PCWP by RHC (HR 1.00, 95% CI 0.96–1.05, *p* = 0.87), TTE (HR 1.30, 95% CI 0.77–2.20, *p* = 0.33), or left ventricular mass by CMR (HR 1.0, 95% CI 0.99–1.01, *p* = 0.67).

In Kaplan–Meier analysis (Figure 4B), participants with simplified CMR-modelled PCWP ≥15 mmHg had worse all-cause mortality over the follow-up period (X^2^ = 4.09, *p* = 0.04), whereas TTE did not demonstrate the prognostic (X^2^ = 2.48, *p* = 0.12).

## 4. Discussion

### 4.1. Main Findings

This study demonstrates that, in a large heterogeneous cohort of patients with suspected or confirmed heart failure, a simplified CMR model can estimate PCWP with good diagnostic accuracy. Increases in pre-load or after-load can result in elevated filling pressures, and since the left atrial area is a marker of the pre-loading condition on the ventricle, the results of this study are physiologically conceivable [8]. This study also demonstrates that a simplified CMR-derived PCWP model has predictive power, suggesting its complementary role alongside echocardiography in assessing diastolic function, particularly in patients with indeterminate echocardiographic assessment. 

### 4.2. Mechanism of Findings

Increases in intracardiac pressure due to cardiac impairment result in re-modelling of both the atrium and ventricle [9]. The findings of this study, where both the left atrial area and left ventricular mass are positively associated with invasively measured PCWP, are consistent with the Frank–Starling mechanism underpinning cardiovascular physiology [10]. 

Dysfunction of the left ventricle is associated with left ventricular hypertrophy, which can be quantified using left ventricular mass [11]. Importantly, LVH is established as an independent marker of poor prognosis [12]. The results of this study are consistent with this phenomenon. Furthermore, the left atrial area as a proxy for left atrial re-modelling in chronic heart failure is thought to result from the cumulative effect of raised cardiac filling pressures and was also found to be associated with RHC-measured PCWP [13]. 

These two main structural pathophysiological changes due to raised LVFP, namely LA dilation (predominantly pre-load-related) and LV hypertrophy (predominantly afterload-related), form the basis of the American College of Cardiology and American Heart Association grading system, commonly used to assess suspected HF patients, and are crucial components of a true physiological model [2]. The CMR model described within this study includes only parameters fundamental to the pathophysiology of HF and is thus simple to translate into clinical practice. 

### 4.3. Clinical Translation

The successful transition of research-based tools into clinical practice hinges on their practical utility for clinicians and patients, which, in the case of CMR-derived PCWP, may rest in identifying patients with abnormal PCWP and haemodynamic guidance of HF therapy [9]. Simultaneously, these tools must enhance clinical workflow without introducing unnecessary complexities. In the case of CMR-derived PCWP assessments, the use of the LAA rather than the LAV is more practical in the clinical setting. 

Whilst the LAV does provide a complete assessment of the left atrium, it does not appear to contribute to a more diagnostically powerful PCWP model than the LAA. So, in cases where precise estimation of atrial volume may be required, such as atrial conditions associated with atria (e.g., congenital heart disease, atrial fibrillation, cardiomyopathies), or in assessing chronicity of diastolic function, the LAV may be the most appropriate metric [14].

Despite providing an enhanced characterisation of the left atrium, there are several limitations to using the LAV within a clinical setting. First, it requires meticulous image acquisition and post-processing, including accurate delineation of the endocardial borders in two views (both the two-chamber and four-chamber views), which can be time-consuming and technically demanding. Second, due to the need for endocardial contouring in multiple views, the measurement of the LAV is associated with increased observer variability. Whilst automated or semi-automated methods can reduce this variability, these tools are not universally available and may vary across clinical settings. If a comprehensive assessment of left atrial structure and function is not required, then clinicians can utilise the practicality and reliability of the left atrial area within pressure assessments, which, in its current form, is most suited to the dichotomisation of PCWP than the exact estimation of the exact value. The LAA measurement is generally simpler and more time-efficient than the LAV, requiring a single view (typically the four-chamber view) and less post-processing, enhancing its practicality within a busy clinical environment.

In comparison to an invasive assessment of filling pressures, CMR offers a non-invasive estimate of LVFP at a lower cost with similar, if not enhanced, prognostic potential. The results of CMR-derived filling pressures can be obtained quickly with good diagnostic accuracy. 

Echocardiography is the first-line non-invasive method of LVFP assessment. It is versatile and cost-effective and can estimate LVFP at the bedside. The reliability of integrated echocardiographic methods for determining raised filling pressures is debatable. This current study highlights the complementary value of CMR in instances where echocardiography is non-diagnostic or indeterminate. The main utility of CMR rests in discrepant cases of echocardiographic assessment rather than accurate estimation of pressure. Further, CMR can provide a wealth of useful diagnostic and prognostic information outside of filling pressure assessment. 

There is a need for further work to explore the role of CMR-derived PCWP assessment in guiding therapy for patients with heart failure. In this situation, precise and accurate estimation of PCWP is less important than a change in response to therapy. This should be tested further. The described method of deriving PCWP is easily applied; there is no need for additional or specialist CMR sequences, as atrial and ventricular quantification are part of routine CMR scans. There is also an additional need to explore other CMR-derived PCWP models, which may improve accuracy, such as myocardial strain and ones that consider a patient’s sex. 

### 4.4. What This Study Adds

This large clinical study expands on previous small research studies involving ill-defined clinical groups. This real-world study utilised standard TTE, which is typically the first imaging investigation of choice in the assessment of cardiovascular structure and function. It is recognised that, since TTE is performed by a range of operators, significant variation in the parameters obtained and the accuracy of such measurements is commonplace. This can affect the estimation of filling pressures. CMR, on the other hand, is associated with much higher repeatability and, therefore, is less subject to variability. We speculate that the CMR model is less susceptible to changes in pre-load conditions than echocardiography since the left ventricular mass is not altered by acute changes in loading conditions. The left atrial area is more susceptible to changes in loading conditions, which adds value in making the model more dynamic and discriminatory. There would be value in testing this suggestion, perhaps in the same patient pre- and post-treatment. We believe that these characteristics of CMR-derived PCWP assessment make it a clinically useful non-invasive imaging test and may reduce the need for invasive assessment. 

The results of this study demonstrate that CMR-derived PCWP is not inferior to invasive PCWP assessment for informing prognosis and may have specific value for cases where assessment with TTE yields an indeterminate probability of ‘raised pressures’. This model, where the left atrial area is used instead of the left atrial volume, highlights how versatile the model is, accommodating several different left atrial measures as a surrogate for pressures. 

### 4.5. Study Limitations

This study’s limitations include its single-centre nature, potentially introducing selection bias due to referrals for right-heart-catheterization (RHC) assessment, which may have contributed to an elevated mean pulmonary artery pressure in the overall population. Caution should be exercised when extrapolating these results to a more diverse and heterogeneous population. Further multi-centre studies would be beneficial to validate the model’s performance across a broader spectrum of patients and healthcare settings. Whilst the LVEF was different between the derivation and validation cohorts, this is unlikely to have influenced the validity of the observed results.

Additionally, the study focused on clinically stable patients with real-world presentations of shortness of breath in outpatient departments, excluding acutely decompensated heart failure (HF) patients requiring intravenous therapy. Acutely decompensated patients often exhibit fluid shifts, hemodynamic instability, and variable filling pressures, which could introduce substantial measurement variability. However, it is worth acknowledging that these patients represent a significant and clinically relevant subgroup within the heart failure spectrum. In light of this, potential future research directions could involve developing specific protocols or methodologies for assessing acutely decompensated patients using CMR, as well as exploring the utility of the CMR-derived PCWP model in guiding their management. 

As a retrospective study within a specific population, the applicability of the proposed algorithm in prospectively recruited, non-selected patients remains untested. Furthermore, limited TTE analysis was conducted, and advanced echocardiographic techniques were not explored, preventing a comparison with our CMR model in these aspects. Future research should explore the use of advanced echocardiographic techniques, such as speckle tracking echocardiography or three-dimensional echocardiography, which may provide more precise measurements of the left atrial area and the left ventricular mass. 

## 5. Conclusions

A simplified CMR model can accurately estimate PCWP in patients with suspected or confirmed heart failure. The study showcases the predictive power of a simplified PCWP model, indicating its potential as a valuable adjunct to echocardiography, particularly in cases with unclear echocardiographic diastolic assessment. This study also emphasises the practicality and efficiency of utilising the left atrial area in PCWP assessment, facilitating a streamlined workflow in clinical settings. Using this revised model, CMR-derived LVFP can inform the risk of decompensation from HF requiring hospitalisation and the risk of composite MACE.

## Figures and Tables

**Table 1:** Patient characteristics, cardiac haemodynamic data, and cardiac magnetic resonance data stratified by right heart catheterisation pulmonary capillary wedge pressure.

	Derivation Cohort (*n* = 708)	Validation Cohort (*n* = 127)	*p*-Value
Age (years)	66.3 ± 13.2	66.0 ± 12.7	0.82
Male sex	295 (42%)	43 (34%)	0.10
Body surface area (m^2^)	1.91 ± 0.25	1.87 ± 0.22	0.22
HFpEF	371 (52%)	71 (56%)	0.47
HFmrEF	29 (4.1%)	3 (2.4%)	0.34
HFrEF	15 (2.1%)	8 (6.3%)	0.008
Other	293 (41%)	45 (35%)	0.20
Heart rate (bpm)	76.0 ± 14.2	75.2 ± 14.7	0.5670
Systolic blood pressure (mmHg)	143.0 ± 26.2	145.6 ± 30.3	0.3174
Diastolic blood pressure (mmHg)	77.5 ± 12.6	78.9 ± 14.0	0.2644
Mean arterial pressure (mmHg)	102.0 ± 17.5	103.9 ± 18.9	0.2854
Mean PCWP (mmHg)	14.0 ± 6.2	13.4 ± 6.3	0.2866
Mean right atrial pressure (mmHg)	10.2 ± 5.8	8.5 ± 5.2	0.0016
Mean pulmonary artery pressure (mmHg)	38.4 ± 13.7	35.4 ± 14.0	0.0265
Systolic pulmonary artery pressure (mmHg)	63.3 ± 24.1	58.3 ± 23.2	0.0317
Diastolic pulmonary artery pressure (mmHg)	22.2 ± 9.6	20.1 ± 10.1	0.0230
Arterial oxygen saturations (%)	94.0 ± 4.3	95.4 ± 3.5	0.0006
Venous oxygen saturations (%)	65.7 ± 8.4	67.2 ± 9,1	0.0866
Cardiac output (L)	5.0 ± 2.0	4.9 ± 1.5	0.5523
Cardiac index (L/min/m^2^)	2.7 ± 1.0	2.7 ± 0.8	0.8798
Left atrial volume (cm^3^)	80.0 ± 43.8	72.5 ± 41.7	0.1239
Left ventricular end-diastolic volume (mL) (indexed)	58.2 ± 18.5	58.1 ± 19.1	0.9784
Left ventricular end-systolic volume (mL) (indexed)	19.6 ± 10.2	20.5 ± 14.9	0.3908
Left ventricular stroke volume (mL) (indexed)	38.6 ± 12.4	37.6 ± 12.4	0.3881
Left ventricular ejection fraction (%) (indexed)	50.6 ± 14.4	56.4 ± 21.4	0.0001
Left ventricular mass (g) (indexed)	78.8 ± 31.6	73.7 ± 29.1	0.0911
Right ventricular end-diastolic volume (mL) (indexed)	45.7 ± 25.6	40.5 ± 22.2	0.0319
Right ventricular end-systolic volume (mL) (indexed)	33.0 ± 13.7	33.2 ± 14.0	0.9438

RHC, right heart catheterisation; PCWP, pulmonary capillary wedge pressure; HFpEF, heart failure with preserved ejection fraction; HFmrEF, heart failure with mid-range ejection fraction; HFrEF, heart failure with reduced ejection fraction.

## Data Availability

The datasets generated and analysed during the current study are not publicly available. Access to the raw images of patients is not permitted since specialised post-processing imaging-based solutions can identify the study patients in the future. Data are available from the corresponding author upon reasonable request.
